# Response to elevated CO_2_ in the temperate C3 grass *Festuca arundinaceae* across a wide range of soils

**DOI:** 10.3389/fpls.2015.00095

**Published:** 2015-02-24

**Authors:** Eric A. Nord, Raúl E. Jaramillo, Jonathan P. Lynch

**Affiliations:** ^1^Department of Plant Science, The Pennsylvania State UniversityUniversity Park, PA, USA; ^2^Department of Biology, Greenville CollegeGreenville, IL, USA

**Keywords:** soil taxonomy, soil orders, elevated CO_2_, *Festuca arundinaceae*, tall fescue

## Abstract

Soils vary widely in mineral nutrient availability and physical characteristics, but the influence of this variability on plant responses to elevated CO_2_ remains poorly understood. As a first approximation of the effect of global soil variability on plant growth response to CO_2_, we evaluated the effect of CO_2_ on tall fescue (*Festuca arundinacea*) grown in soils representing 10 of the 12 global soil orders plus a high-fertility control. Plants were grown in small pots in continuously stirred reactor tanks in a greenhouse. Elevated CO_2_ (800 ppm) increased plant biomass in the high-fertility control and in two of the more fertile soils. Elevated CO_2_ had variable effects on foliar mineral concentration—nitrogen was not altered by elevated CO_2_, and phosphorus and potassium were only affected by CO_2_ in a small number of soils. While leaf photosynthesis was stimulated by elevated CO_2_ in six soils, canopy photosynthesis was not stimulated. Four principle components were identified; the first was associated with foliar minerals and soil clay, and the second with soil acidity and foliar manganese concentration. The third principle component was associated with gas exchange, and the fourth with plant biomass and soil minerals. Soils in which tall fescue did not respond to elevated CO_2_ account for 83% of global land area. These results show that variation in soil physical and chemical properties have important implications for plant responses to global change, and highlight the need to consider soil variability in models of vegetation response to global change.

## Introduction

Substantial attention has been given to the effects of elevated CO_2_ concentration on plant growth and physiology (Körner, 2006), reflecting concern about the performance of both cultivated and wild plants in future climates characterized by elevated CO_2_ (IPCC, 2007a). Studies of the responses of crops and ecosystems to elevated CO_2_ (Körner, 2006; Ziska and Bunce, 2006, 2007) often report increased growth and use efficiency of nitrogen and water when nutrient availability is optimal or near optimal (Woodward et al., 1991; Bunce, 2004). Though the majority of studies consider elevated CO_2_ in isolation, plant responses to elevated CO_2_ may be affected by other environmental factors, including soil properties (Diaz et al., 1993; Bassirirad et al., 2001; Spinnler et al., 2002; Lynch and St. Clair, 2004, 2010; Fay et al., 2009, 2012a).

When suboptimal nutrient availability has been considered (generally as deficiency of either N or P), a commonly observed response is that a limited supply of N or P leads to a reduction in photosynthetic rates and foliar N concentrations and increased concentrations of non-structural carbohydrates (NSC) (van Noordwijk et al., 1998; Gifford et al., 2000; Gifford, 2004). Few studies have considered multiple nutrient stresses (deficiencies and/or toxicities) in conjunction with elevated CO_2_ (Körner, 2006), and our understanding of the mechanisms behind the interaction of soil characteristics with elevated CO_2_ is far from complete (Lynch and St. Clair, 2004). In contrast to research with crops and model plants, forestry and ecological research has considered the effects of elevated CO_2_ in natural soils without amendments or fertilizers. Such studies generally indicate multiple and complex limitations, mostly of edaphic origin, that trees face under elevated CO_2_ (Bucher-Wallin et al., 2000; Bassirirad et al., 2001; Egli et al., 2001; Poorter and Perez-Soba, 2001; Spinnler et al., 2002).

Natural soils vary widely across terrestrial ecosystems; the USDA soil taxonomy system addresses this variability by classifying soils into 12 orders based on factors related to soil formation (Wilding, 2000; Table S1). These are further divided into 64 suborders and additional sub-categories based on climatic and edaphic modifiers (Soil Survey Staff, 1999). One purpose of such taxonomic systems is to optimize land use over the range of soils so as to maximize productivity and sustainability (Driessen and Konijn, 1992). The variability described by soil taxonomy may also be useful in understanding climate change effects, such as the effect of elevated CO_2_ on plant growth, but there has been little effort made to test whether, and to what extent, the effects of elevated CO_2_ on plant growth depend on soil taxonomic variation.

A literature search performed on combinations of “Elevated CO_2_” and edaphic and soil taxonomy terms produced a relatively small number of hits (Table S2 in Supplemental Materials)– only 57 unique publications were returned by searching “Elevated CO_2_ AND soil type” and “Elevated CO_2_ AND (any USDA soil order)” on Web of Science. Further analysis of topics for the 57 unique publications highlight the scarcity of consideration of the range of possible soil impacts on CO_2_ responses (Tables S3, S4 in Supplemental Materials). Only 21 of these publications report results from an experiment with two or more soils and elevated CO_2_, and the maximum number of soils considered was three. Furthermore, these 21 publications report on only 6 unique experiments, and 16 of the 21 reports and 3 of the 6 unique experiments relate to woody plants. Although several authors have noted the importance of soils in determining plant responses to elevated CO_2_ (Bassirirad et al., 2001; Poorter and Perez-Soba, 2001; Spinnler et al., 2003; Fay et al., 2009, 2012a), it appears that no attempt has been made to test the extent to which plant responses to elevated CO_2_ vary across the natural variability of soils.

As noted above, work on woody plants may be more advanced than work in non-woody plants in this area. Watanabe et al. (2013) reported no CO_2_ × soil interaction on photosynthetic traits of hybrid *Larix* grown for two seasons on a fertile forest soil and an infertile volcanic ash soil. In contrast, Spinnler et al. (2002) found that while in *Picea* there was no CO_2_ × soil interaction on biomass, in *Fagus* elevated CO_2_ only stimulated growth in a more fertile calcareous soil, and actually suppressed growth on an acidic soil. In the same system it was found that responses to elevated CO_2_ may differ between root and shoot (Sonnleitner et al., 2001). Such differences may have ecosystem consequences; another report on the same system showed that the acidic soil increased its carbon content to a much greater degree than the calcareous soil, even though it supported much less biomass (Hagedorn et al., 2003). Interestingly, while biomass responses of *Picea* and *Fagus* in this system were strongest in the early years of this 4 year study, plant water relations still responded to elevated CO_2_ in a soil-dependent manner (Bucher-Wallin et al., 2000). In contrast, another study showed no effect of elevated CO_2_ on stomatal conductance or leaf hydraulic conductivity in *Betula* or *Quercus* grown on two contrasting soil, though responses to CO_2_ differed between sun and shade leaves (Eguchi et al., 2008).

The best examples for non-woody plants are a series of reports on experiments carried out with monoliths of three soils that were exposed to a CO_2_ gradient from sub- to super-ambient. In this system early growth of *Panicum virgatum* was enhanced by elevated CO_2_ but not regrowth after clipping. An interaction of Soil × CO_2_ was seen for soil moisture but not for annual net primary productivity (Fay et al., 2012b). Another study using this system with constructed prairie plant communities found that aboveground biomass response to CO_2_ was greatest on soils with greater plant available water (a Mollisol and an Alfisol) and was reduced on a heavy clay soil (a Vertisol) with lower plant available water (Fay et al., 2012a). Another report on this system showed that in the Mollisol forbs responded more strongly to elevated CO_2_ than grasses, though grasses were stimulated by increasing CO_2_ in all three soils (Polley et al., 2012).

Grasses (Poaceae) include cereal crops that provide over half of the calories and protein consumed by humans (Cordain, 1999) and are the principle vegetation of the 30% of global land area occupied by natural grasslands (Bartholome and Belward, 2005; Lambin and Geist, 2006). Grasses represent a large, variable group of plants that are successful in many environments and that have evolved several mechanisms to adapt to extreme soil conditions (Marschner, 1998).

In order to begin to characterize the effects of the wide range of soils on plant responses to elevated CO_2_ we grew tall fescue (*Festuca arundinaceae* Schreb.), a temperate C3 grass, in elevated and ambient CO_2_ on 13 different soils, representing ten of the twelve soil orders, and a high fertility control, and assessed plant growth, mineral acquisition, gas exchange, and non-structural carbohydrate accumulation.

## Materials and methods

### Experimental setup

Tall fescue was grown in eight Continuous Stirred Tank Reactors (CSTRs; mylar covered cylindrical steel frames approximately 2 m in diameter and 2 m tall with a continually rotating stirring paddle near the top to ensure even mixing of the atmosphere; Heck et al., 1975) in a greenhouse at the Pennsylvania State University (40°85'N, 77°83'W). The CSTRs were covered in transparent mylar and fitted with a positive pressure ventilation system that provided an airflow of 1 L per minute to each CSTR. Each CSTR was equipped with an external overhead 1000 watt HID Lamps for supplemental light; maximum light intensity at plant level averaged 350 μmol PAR s^−1^ m^−2^ (This relatively low lighting intensity reflects both the attenuation of solar radiation through both greenhouse roof and the mylar covering of the CSTRs and the difficulty of controlling heat load from the HID lighting supplied to each CSTR).

The eight CSTRs were grouped into four pairs, with one of each pair receiving near ambient (400 ppm CO_2_, which was near the ambient level in the greenhouse) and the other receiving elevated (800 ppm CO_2_, corresponding to the IPCC's “worst case” A1F1 scenario for mid; IPCC, 2007b). Elevated CO_2_ was maintained by bleeding 99.8% dry CO_2_ from a pressurized tank via a needle valve into a manifold from which four valves controlled the flow of CO_2_ to each of the elevated CO_2_ CSTRs. These valves were adjusted daily to maintain the target CO_2_ concentration. CO_2_ concentration (measured with a Li-Cor 6262 infrared gas analyzer connected to a multiplexing pump), temperature, photosynthetically active radiation (PAR), and relative humidity for each CSTR were recorded every 16 min. CO_2_ concentrations were relatively stable, with mean values (±1 standard deviation of 790 ± 14 ppm for Elevated CO_2_ and 399 ± 11 ppm for near ambient CO_2_.

### Planting

Soil samples representing 10 taxonomic orders (Table 1) were obtained from Puerto Rico, Ecuador and the U.S. (Alaska) during 2005 and 2006. In each location we collected from areas with no known history of fertilizer use. Soils were air dried and transported to University Park, PA., where they were kept in refrigerated storage (6°C) until the experiment began in January 2007, when the soils were sieved (2 mm) to exclude gravel and organic debris, and eight pots (400 ml volume) were filled with each soil type. Pots were also prepared with a high-fertility control treatment (“CTR”), consisting of a standard horticultural medium based on peat and vermiculite (Sunshine Mix #3, Sun Gro Horticulture, Bellevue, WA) amended with a complete slow-release fertilizer (Osmocote 14-14-14 in a rate of approximately 3 g per pot; Scotts Miracle-Gro, Marysville, OH).

**Table 1:** **Properties of soils used in the study**.

**Soil ID**	**Order (suborder)**	**Origin**	**pH**	**Total N (%)**	**P (ppm)**	**K (ppm)**	**Mg (ppm)**	**Ca (ppm)**	**Zn (ppm)**	**Cu (ppm)**	**S (ppm)**	**Clay%**	**Silt%**	**Sand%**
ALF	Alfisol (Udalf)	PR	6.4	0.33	7	210	147	2065	3.6	4.4	46	15.3	22.3	62.3
AND	Andisol (Aquand)	EC	5.4	0.47	12	148	73	439	3.7	5.9	29.3	1.06	28.4	70.5
ARD	Aridisol	EC	7.9	0.09	71	182	633	2582	4.2	9.4	36.2	8.06	41.5	50.4
	–													
INC1	Inceptisol (Tropent)	EC	5.6	tr	7	86	168	768	3.5	3.1	19.6	0.45	5.46	94.1
INC2	Inceptisol (Udept)	PR	4.7	0.05	1	84	17	120	0.3	3.8	216	NA	NA	NA
GEL	Gelisol	AK	7.7	0.19	9	50	164	5490	NA	NA	NA	12.1	59.7	28.2
	–													
MOL	Mollisol (Ustol)	PR	7.5	0.16	200	689	497	5001	4.5	10.2	22.4	30.1	31.4	38.5
OXI1	Oxisol (Ustox)	PR	7.7	0.21	11	280	124	2987	1.7	5.2	17.7	26.0	29.2	44.8
OXI2	Oxisol (Udox)	PR	5.2	0.15	1	44	57	189	0.7	1.4	285	4.1	11.6	84.3
OXI3	Oxisol	EC	5.8	0.26	9	28	52	522	1.4	2	18.9	13.0	29.1	57.9
	–													
SPO	Spodosol (Orthod)	PR	7.3	0.19	20	33	125	3566	19.4	13.6	29.5	1.56	11.1	87.3
ULT	Ultisol (Humult)	PR	5.4	0.29	14	545	225	1248	2.6	6.4	44.3	20.8	33.2	46.1
VRT	Vertisol (Ustert)	PR	6.8	0.15	10	150	1737	5016	1.4	8	23.3	22.9	29.7	47.4

*PR, Puerto Rico; EC, Ecuador; AK, Alaska (United States); NA, Not available; tr, trace values*.

Approximately 20 seeds of tall fescue (cultivar Kentucky 31; SeedLand, Inc. Wellsborn, FL) were broadcast on the surface of each pot on Jan 23, 2007. The pots were covered in clear plastic until germination, after which pots were thinned to 15 plants pot^−1^. 14 pots (the 13 soils plus the fertile control) were randomized within each of the eight CSTRs, for a total of 112 pots. Pots were irrigated manually with distilled water every day.

### Data collection

The presence of the endophytpe *Neotyphodium sp*. was assessed from a sample of 100 seeds and two growing tillers per pot (from two replicates), using a commercial immunoblot detection kit (Agrinostics Ltd. Co., Watkinsville, GA).

Leaf photosynthesis (A_max_, area basis) of a young, fully expanded leaf in one plant per pot was determined on weeks 8 and 10 at mid-day with a LI-6400 portable photosynthesis system (Li-Cor, Lincoln, NE) with leaf temperature set to 20°C. Measurements were made at 600, 800, and 1000 μmol m^−2^ s^−1^ PAR, after measurements at 200–1000 μmol m^−2^ s^−1^ PAR on a subset of plants showed that maximum photosynthetic rate occurred in the 600–1000 μmol m^−2^ s^−1^ range. Also at week 10 the net pot CO_2_ exchange (including soil) was measured with a Li-6200 Infrared Gas Analyzer system (Li-Cor, Lincoln, NE) using a 12 liter chamber in which the whole plant and pot were enclosed for 2 min. During these measurements temperature ranged from 25 to 28°C, and light (PAR) ranged from 320 to 380 μmol m^−2^ s^−1^, reflecting the ambient growing conditions.

Above-ground tissue was harvested following CO_2_ exchange measurement. Immediately following shoot excision, the same method was used to measure the amount of respiration from the roots and soil. Canopy CER was estimated as the difference between the net pot CO_2_ exchange and root + soil CO_2_ exchange.

Excised shoots were divided into three samples. The first sample (~5 g fresh weight) was frozen at −80°C. These were later processed to quantify ethanol soluble sugars and starch from approximately 100 mg of ground tissue with the enzyme-coupled colorimetric method described by Hendrix (1993).

All remaining above-ground tissue was oven dried at 60°C for 48 h to determine total dry weight. A sample of about 0.2 g of dried and ground leaf tissue was digested in a microwave (Miller, 1998). The diluted (250:1) extract was analyzed in a Varian Induced Couple Mass Spectrophotometer (Varian Inc., Palo Alto CA) to determine the content of: phosphorus, potassium, calcium, magnesium, manganese, iron, copper, boron, aluminum, zinc and sodium. A second sample of dried and ground tissue was analyzed using a Perkin-Elmer EA2400 elemental analyzer with combustion and reduction columns to determine cabon and nitrogen content.

### Statistical analysis

The data were analyzed with a split-plot design. The four pairs of CSTRs were treated as blocks, with CO_2_ as the main plot factor and soil the subplot factor. Data were analyzed in R (R Core Team, 2014) using *lme* (Pinheiro et al., 2014), to fit linear mixed effects models with block and CSTR as random effects (The model was “response ~ CO2 + Soil + Soil:CO2, random = ~1|Block/CSTR”), and residuals were checked to ensure that regression assumptions were not violated. For A_max_, the mixed effect model included all saturating levels of PAR with PAR as a random effect (The model was “Amax ~ CO2 + Soil + Soil:CO2, random = ~1|Block/CSTR/PAR”). Pair-wise Tukey comparisons for the effect of CO_2_ in each soil were obtained using *multcomp* (Hothorn et al., 2008).

Given the large number of variables measured (31) Principal component analysis (PCA) was used to characterize the principal sources of variability in the data. PCA was carried out for all observations with growth, photosynthesis, soil analysis and leaf mineral concentrations as variables using *prcomp* (R Core Team, 2014).

## Results

About 40% of the seeds and 95% of all tillers tested positive for the *Neotyphodium* endophyte, and *Neotyphodium* colonization did not differ among soils or CO_2_ treatments.

**Figure 1:**
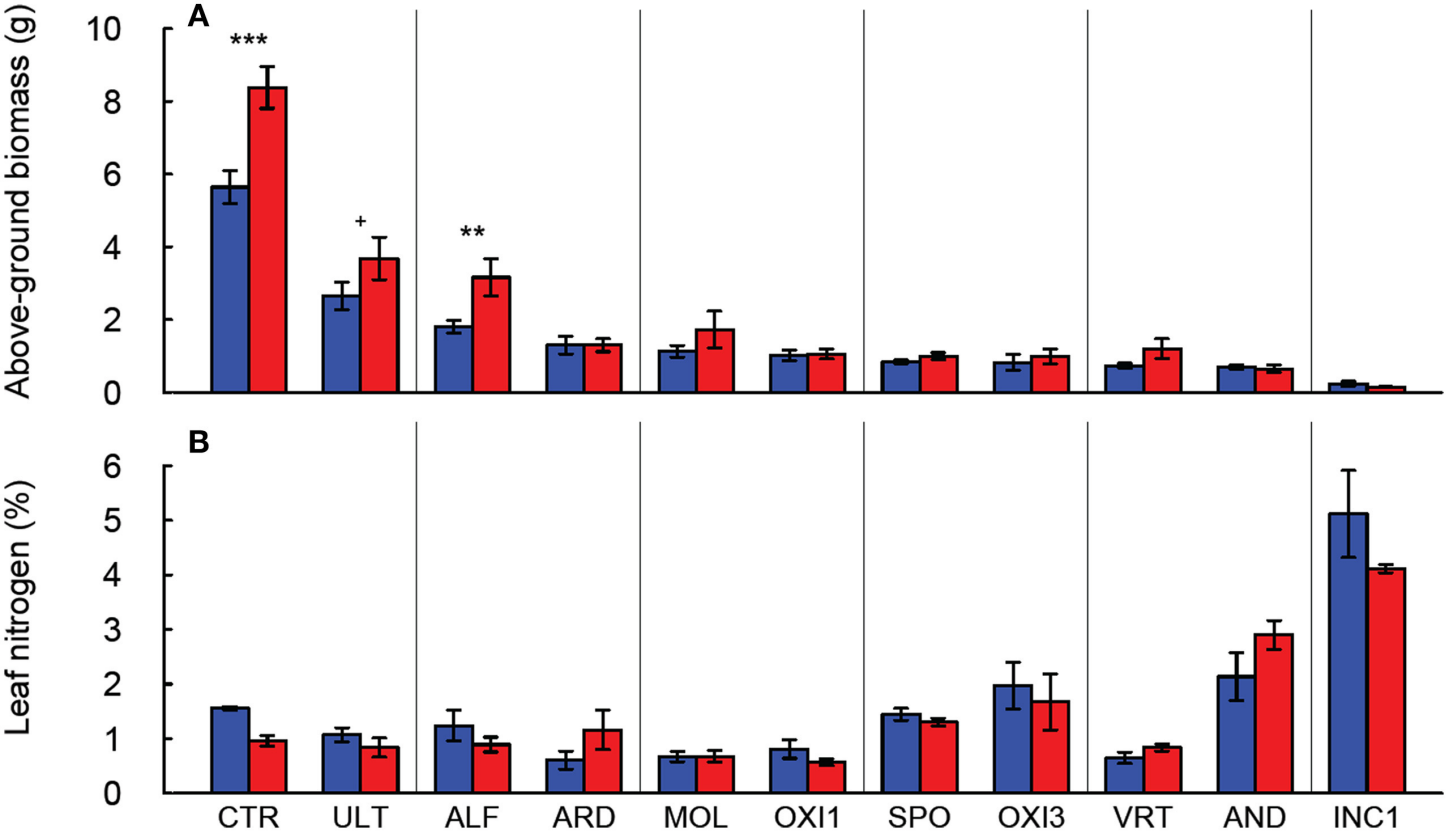
**Production of above-ground biomass (A) and leaf nitrogen content (B) of *Festuca arundinacea* grown in 12 different soils and a high-fertility control under elevated (800 ppm; red) and ambient (400 ppm; blue) atmospheric CO_2_**. Mean of four replicates ± one standard error shown. Significance indicated based on pair-wise Tukey comparisons. The soils are ordered by decreasing biomass in ambient CO_2_. ^+^*p* = 0.1–0.05; ^**^p = 0.01–0.001; ^***^p < 0.001.

Of the soils used in this experiment, only INC2 did not support any germination of tall fescue. Plants grown in GEL, HIS and OXI2 exhibited severe reductions (>97%) in biomass compared to CTR, and did not produce sufficient tissue for all analyses to be completed. They were therefore eliminated from most of the subsequent analyses. The remaining soils produced 50–80% less shoot biomass than CTR, indicating significant differences among soils (Table 2). These differences were especially notable under elevated CO_2_, where biomass of CTR increased by about 60% (Figure 1A). Elevated CO_2_ significantly increased plant biomass in the CTR, ULT and ALF (by approximately 20–40%), but did not increase biomass in the other soils (Figure 1A).

**Table 2:** **Summary of analysis of variance results of biomass, mineral content, and gas exchange parameters for Festuca arundinacea var**.

		**Biomass**		**A_max_**		**Shoot CER**	**Pot Resp**.
Source	df	*F*-value	df	*F*-value	df	*F*-value	*F*-value
CO_2_	1/3	6.98^*^	1/3	7.71+	1/3	1.01	7.29+
Soil	13/77	117^***^	10/208	15.5^***^	10/60	10.8^***^	19.1^***^
CO_2_ × Soil	13/77	5.50^***^	10/208	6.57^***^	10/60	0.903	2.24^*^
		**N**		**C:N**		**Starch**	**Sucrose**
CO_2_	1/3	2.12	1/3	1.13	1/3	15.8^*^	3.01
Soil	12/59	25.5^***^	10/55	7.50^***^	9/54	2.05+	2.79^**^
CO_2_ × Soil	12/59	1.32	10/55	0.696	9/54	1.25	0.711
		**P**	**K**	**Ca**	**Mg**	**Mn**	
Source	df	*F*-value	*F*-value	*F*-value	*F*-value	*F*-value	
CO_2_	1/3	8.43+	4.51	18.9	15.8^*^	11.6^*^	
Soil	10/56	55.1^***^	20.7^***^	72.6^***^	26.7^***^	126^***^	
CO_2_ × Soil	10/56	3.26^**^	3.70^***^	3.08^**^	1.78+	1.75+	
		**Al**	**Fe**	**B**	**Na**	**Zn**	
CO_2_	1/3	0.00111	0.0181	0.00137	3.10	7.75+	
Soil	10/56	4.68^**^	16.4^***^	13.5^***^	23.4^***^	104^***^	
CO_2_ × Soil	10/56	0.423	1.77+	0.624	1.72+	3.60^**^	

Kentucky 31 grown in ambient (400 ppm) and elevated (800 ppm) CO2 in 13 different soils plus a high-fertility control.^*^

^*^ Not all soils produced sufficient biomass for all analyses, leading to differences in degrees of freedom between analyses. Degrees of freedom (df) printed to the left of each variable and listed as numerator df/denominator df. Pot Resp. = Root + Soil CO2 exchange rate. Significance denoted as:

+ p = 0.1–0.05;

* p = 0.05–0.01;

** p = 0.01–0.001;

*** *p < 0.001*.

In the soils used in this experiment elevated CO_2_ did not significantly change leaf nitrogen concentration (*p* = 0.323; Figure 1B, Table 2), though there were differences in leaf N associated with the soils (*p* < 0.001), as would be expected. Furthermore, the C:N ratio was not altered by CO_2_ or the CO_2_ × soil interaction (*p* > 0.300, data not shown), though it did differ among the soils (*p* < 0.001, Table 2).

**Figure 2:**
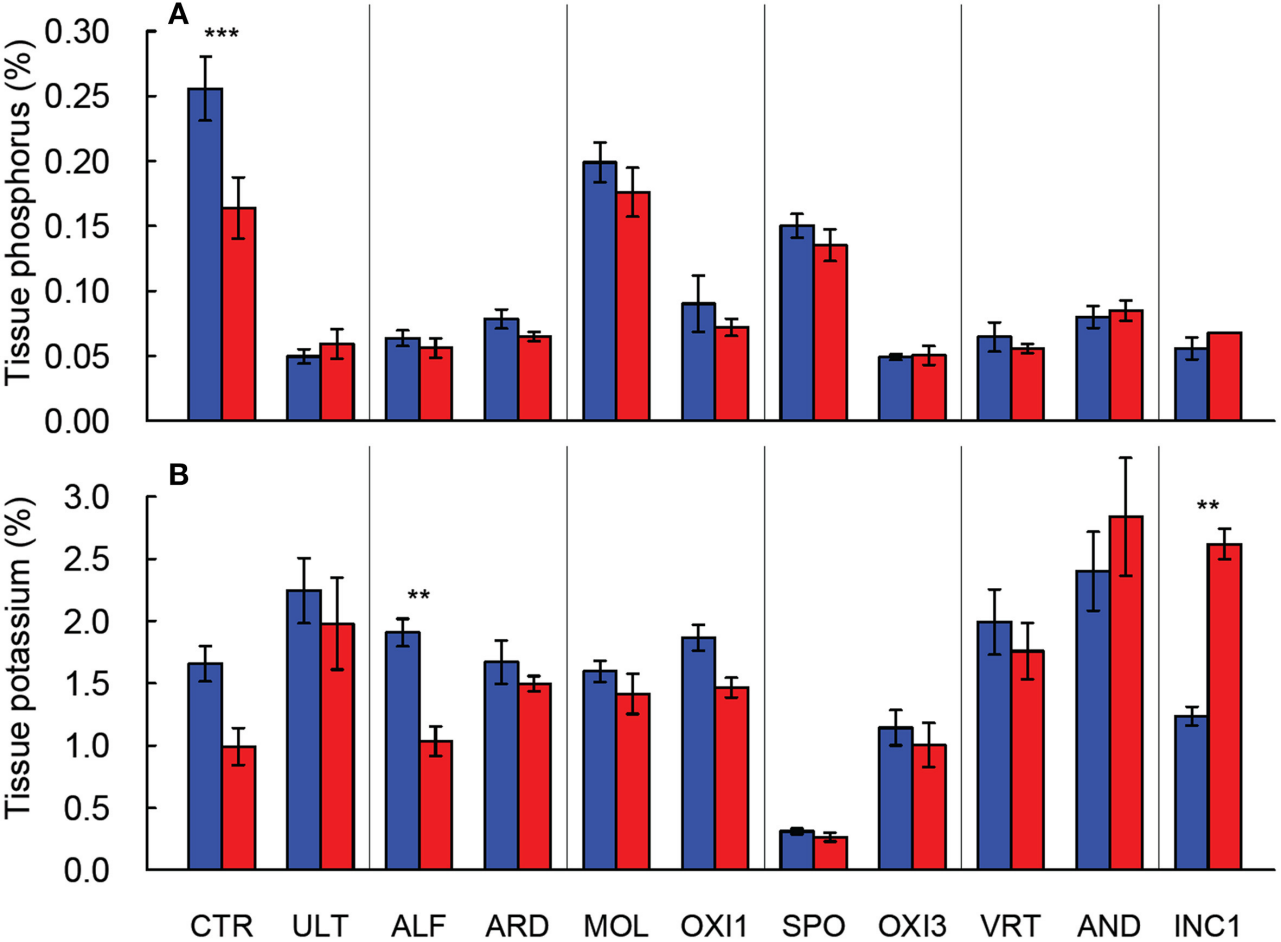
**Leaf P (A) and K (B) concentration in shoots of *Festuca arundinacea* grown in 10 soils and a high fertility control under elevated (800 ppm; red) and ambient (400 ppm; blue) CO_2_**. Mean of four replicates ± one standard error shown; lack of error bar indicates missing data points. The soils are ordered by decreasing biomass in ambient CO_2_, as in Figure 1A. ^**^p = 0.01–0.001; ^***^p < 0.001.

Leaf phosphorus concentration declined approximately 30% with elevated CO_2_ in CTR, while in the remaining soils phosphorus concentration changed only marginally (*p* = 0.062; Figure 2A). Leaf potassium concentration was reduced by 40% under elevated CO_2_ in ALF soil, and increased by 100% in INC1, but was not affected in the remaining soils (*p* > 0.650; Figure 2B). Leaf K concentration was dramatically lower in SPO than in CTR, but the remaining soils produced leaf K values within about ±20% of CTR (Figure 2B).

The ANOVA analysis for leaf mineral concentrations found highly significant differences (p < 0.001) for the soil effect for all leaf nutrient concentrations measured. However, the results for CO_2_ and the soil × CO_2_ interaction were less consistent. For example the soil × CO_2_ interaction was significant for P, K, Ca, and Zn, and only marginally significant for Mg, Mn, and Fe. (Table 2). Elevated CO_2_ increased content of calcium in INC1 and SPO (*p* < 0.001), of magnesium in CTR (*p* = 0.038) and OXI3 (*p* = 0.003), of manganese in OXI3 (*p* < 0.001); of sodium in SPO (*p* < 0.001); and of zinc in CTR (*p* < 0.001) and SPO (*p* = 0.033).

**Figure 3:**
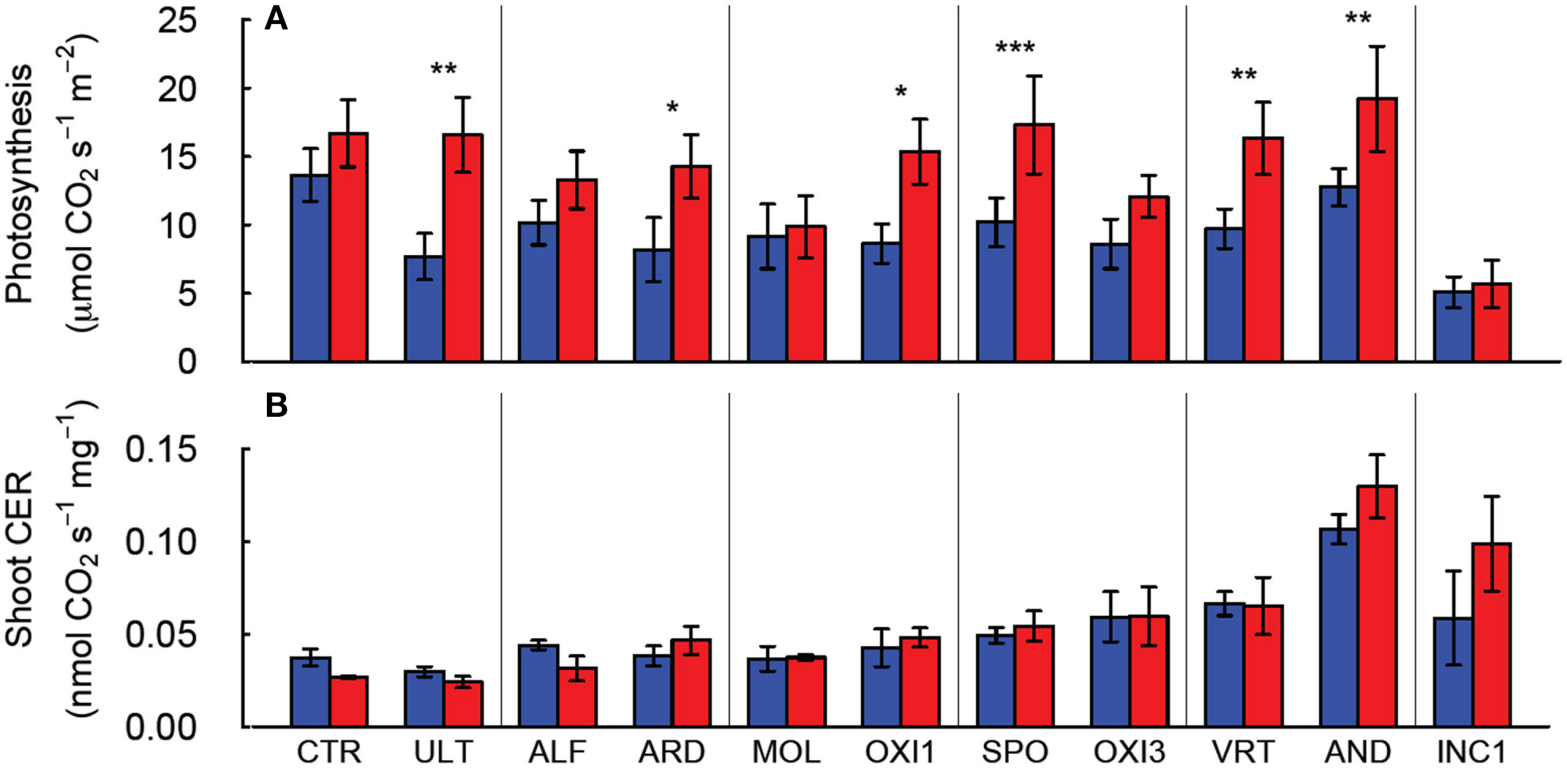
**Leaf photosynthesis of individual leaves (A) and net carbon exchange rate (CER) for the shoot (B) of *Festuca arundinacea* grown in 10 soils, plus a high-fertility control under elevated (800 ppm; red) and ambient (400 ppm; blue) atmospheric CO_2_**. Mean of four replicates ± one standard error shown. The soils are ordered by decreasing biomass in ambient CO_2_, as in Figure 1A. ^*^p = 0.05–0.01; ^**^p = 0.01–0.001; ^***^p < 0.001.

Elevated CO_2_ increased leaf photosynthesis (A_max_, μmol CO_2_ s^−1^ m^−2^ leaf area) in most of the soils, but not in CTR, ALF, MOL, and OXI3, reflecting a significant soil × CO_2_ interaction (*p* < 0.001; Table 2, Figure 3A). Among the soils A_max_ in ambient CO_2_ was highest in CTR and AND in ambient and elevated CO_2_ respectively and lowest in INC1 (both CO_2_ levels; Figure 3A;. Table 2).

Aboveground carbon exchange rate (Shoot CER; nmol CO_2_ s^−1^ mg^−1^ DW canopy) did not differ significantly between elevated and ambient CO_2_ overall (*p* = 0.389), but varied between soils (*p* < 0.001), and there was no interaction between CO_2_ and soil (Table 2, Figure 3B). Root + soil CO_2_ exchange rate (nmol CO_2_ s^−1^) increased in CTR, ALF, ARD, and MOL, but not in the others (Figure S1), leading to a significant Soil × CO_2_ interaction (*p* = 0.027; Table 2).

Combining CO_2_, biomass production, mineral content and photosynthesis variables we created a matrix of 29 variables and 76 observations after records with missing data were excluded (mostly soils in which insufficient biomass was produced for all analyses to be completed; HIS, GEL, INC2, and OXI2) were excluded. Since % Sand, % Silt, and % Clay sum to 100%, we excluded % Sand from this analysis. Similarly, since CEC is reflective of soil Ca and Mg, we excluded CEC.

**Figure 4:**
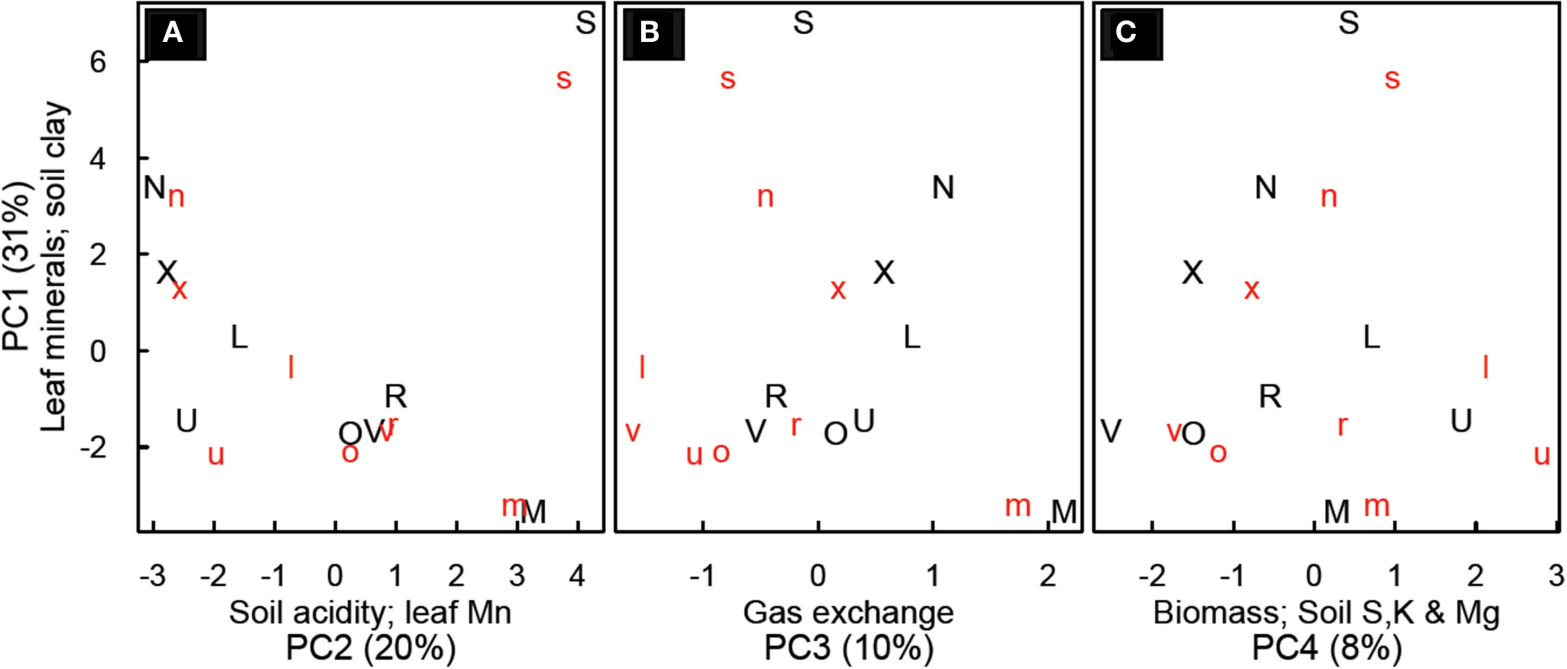
**Principal component analysis for *Festuca arundinacea* grown in 10 soils, plus a high-fertility control at two CO_2_ levels**. Principal component (PC) 1 vs. PC2 **(A)**; PC1 vs. PC3 **(B)** and PC 1 vs. PC4 **(C)**. Lower-case red letters represent elevated CO_2_ (800 ppm) and upper-case black letters ambient CO_2_ (400 ppm). Axes are labeled with the principal component, the variables most strongly related to that principle component, and (in parentheses) the percent of variability explained by that principle component. The symbols represent the soils as follows: L, Alfisol; N, Andisol; R, Aridisol; M, Mollisol; O, Oxisol1; X, Oxisol3; S, Spodosol; U, Ultisol; V, Vertisol.

Principal components analysis yielded four principal components (PC) which explained about 65% of the variability in the data (Figure 4). PC1 (29% of variability) was most strongly influenced by foliar mineral concentrations (Zn, Cu, Mg, Ca, and Na) and soil clay content (minerals declining with increasing clay). PC2 (19% of variability) was most influenced by soil copper, soil calcium, soil pH foliar P and foliar Mn concentrations (all others decrease when foliar Mn increases). PC3 (9% of variability) was most strongly influenced by gas exchange (leaf photosynthesis and stomatal conductance) and foliar Al, P, Fe and C (photosynthesis increasing with decrease in foliar minerals). PC4 (8% of variability) was most strongly influenced by above-ground biomass, sucrose, soil S, soil K, and Mg (with Mg decreasing when others increase; Table S5).

**Figure 5:**
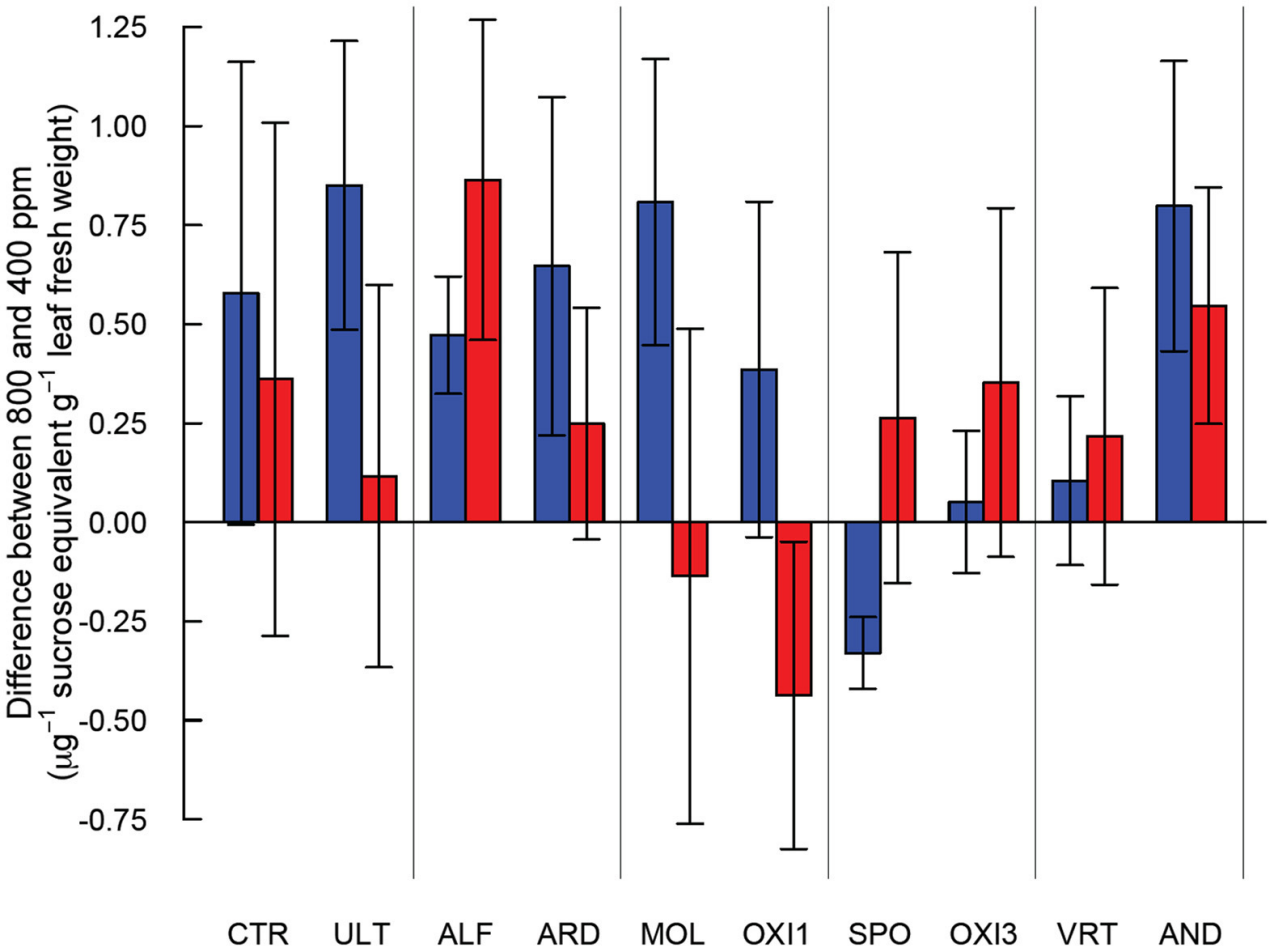
**Differences in starch (blue) and sucrose (red) content between *Festuca arundinacea* grown under elevated and ambient atmospheric CO_2_ in 10 soils, plus a high-fertility control**. The soils are ordered by decreasing biomass in ambient CO_2_ as in Figure 1A, and those which produced insufficient biomass for analysis are not shown.

High and low molecular weight NSC (starch and sucrose respectively) responded differently in this experiment (Table 2). Starch concentrations increased under elevated CO_2_ (*p* = 0.029), but sucrose concentrations were not affected by CO_2_ (*p* = 0.181). Levels of NSC were influenced by the different soils (*p* = 0.051 and 0.009 for starch and sucrose respectively; Table 2). However, the effect of elevated CO_2_ on NSC did not depend on soil type (*p* = 0.288 and 0.697 for starch and sucrose respectively; Table 2, Figure 5).

**Figure 6:**
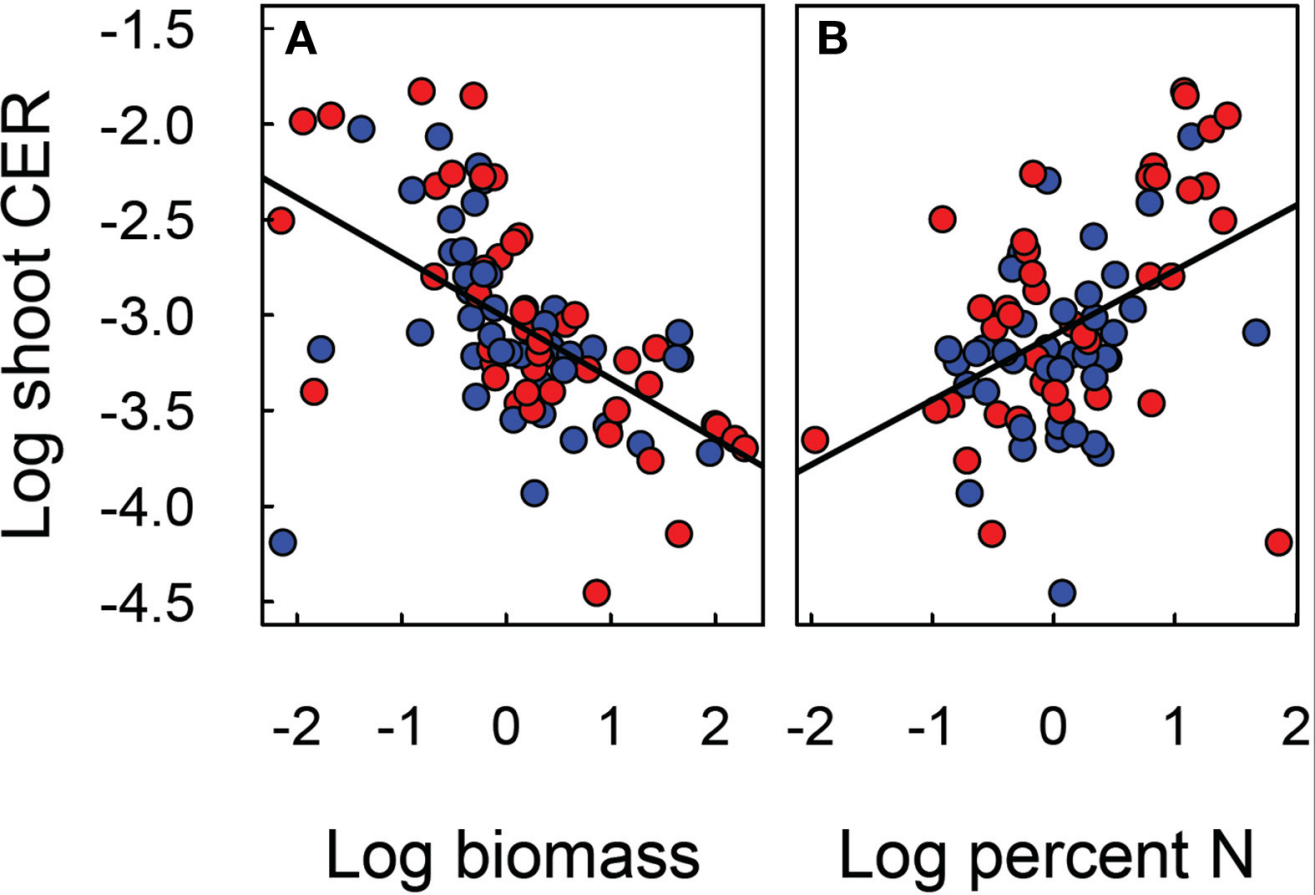
**Relationship of shoot CER to shoot biomass (A) and nitrogen content (B) of *Festuca arundinacea* grown in 10 soils, plus a high-fertility control under elevated (800 ppm; red) and ambient (400 ppm; blue) atmospheric CO_2_**. The solid line indicates N:P ratio of 13 and the dotted lines ratios of 9 and 19. The solid line illustrates the linear regression model fit to the data in each panel.

Maximum photosynthetic rate (A_max_) did not show a strong correlation with foliar N (Figure 6A). Furthermore, the relationship between A_max_ and foliar N differed between soils (*p* = 0.0008) and marginally with CO_2_ (*p* = 0.079). A graphical analysis (based on non-overlap of SE ellipses, Figure 6A) suggests that for OXI1, ULT, SPO, CTR, and AND A_max_ may not be strongly related to foliar N. The tukey test of pair-wise differences confirms this for OXI1 and ULT (*p* = 0.006 and 0.062).

Analysis of N:P ratios (Figure 6B, Table 2) shows a strong effect of soil (*p* < 0.001) but no effect of CO_2_ (*p* = 0.410), or their interaction (*p* = 0.131). Three rough groupings of soils are differentiated here. CTR and MOL, with a low N:P ratio, INC1, AND, and OXI3, with a high N:P ratio, and ARD, ALF, OXI1, SPO, ULT, and VRT with intermediate values.

## Discussion

In this experiment we evaluated the effect of elevated CO_2_ on the growth and physiology of *Festuca* encountering different chemical and physical soil characteristics presented by soils from 9 of the 12 soil orders, spanning the global range of soil variability. The effect of elevated CO_2_ on growth, photosynthesis, and leaf chemistry depended on the soil in which the plants were grown. Since we provided adequate irrigation, we assume that the responses we observed mostly reflect the ability of *Festuca* to acquire nutrients from the different soils under contrasting CO_2_ regimes.

Of the 13 soils used in this experiment four soils either failed to permit germination or to produce sufficient tissue for all of our assays. These are extreme soils for which *Festuca*, despite its wide range of adaptability (Malinowski and Belesky, 2000; Rahman and Saiga, 2007), is apparently not well adapted. These soils include the two soils with the lowest phosphorus values (INC2 and OXI2) and the lowest pH of all the soils (INC2).

Although *Neotyphodium* is restricted to the aerial parts of the plant, its presence has been related to increased tolerance of fescue to several edaphic stresses, including P and Ca, (Malinowski et al., 2000) though the effect of endophyte infection may depend on plant genotype and soil (Rahman and Saiga, 2007). We found no effect of soil or CO_2_ on colonization of *Neotyphodium* in fescue; we therefore do not expect *Neotyphodium* colonization to favor specific soil treatments (Malinowski and Belesky, 2000).

We found large differences between soils in the effects of elevated CO_2_ on *Festuca*. First, there was a large increase in biomass under elevated CO_2_ on soils where biomass was greater under ambient CO_2_, in contrast to the lack of stimulation seen in soils that supported less biomass production in ambient CO_2_. To some extent, this may simply indicate that soils where *Festuca* grows well support a greater increase in biomass in elevated CO_2_; i.e., there may be a correlation between biomass increase in elevated CO_2_ and biomass in ambient CO_2_. This relationship is significant, but only explains 44% of the variability in CO_2_ response (*p* < 0.001, *R*^2^ = 0.44). This suggests that other factors may also be important.

Our results generally agree with previous reports of the lack of response to increased CO_2_ under nutrient-limited conditions (Poorter and Perez-Soba, 2001; Ziska and Bunce, 2006). Differences in leaf elemental concentration highlight a second important response; some soils have inherent low levels of N, P and K, and plants do not accumulate these elements to sufficient levels, and therefore may experience limitation by these elements. For example plants grown in Spodosols had very low foliar K (Figure 2B), suggesting K limitation as a possible cause for the low biomass production (Figure 1A) in this soil. In some cases elevated CO_2_ led to the accumulation of non-limiting elements. For example under elevated CO_2_ plants in AND and INC1 had higher levels of foliar N than CTR or any other more fertile soil; plant growth in these soils was apparently not limited by nitrogen, but the very low biomass of these plant points at some limitation (Figure 1).

The carbon exchange rate for whole shoot and leaf photosynthesis (μmol CO_2_ m^−2^ leaf s^−1^) also showed contrasting results (Figure 3). While leaf photosynthesis (A_max_) showed the expected increase in response to elevated CO_2_ in six of the soils, shoot CER was not altered by elevated CO_2_. The difference between leaf and canopy photosynthetic responses to elevated CO_2_ was not a simple product of changes in biomass as shoot CER was normalized by biomass. There was a significant negative relationship (log-linear) between shoot CER and shoot biomass (Figure 6A, *p* < 0.001, *R*^2^ = 0.30). However, shoot CER was positively related (log-linear) with leaf N (Figure 6B, *p* < 0.001, *R*^2^ = 0.17). There are reports of growth dilution of leaf N by elevated CO_2_ (Luo et al., 1994; Idso and Idso, 2001; Taub and Wang, 2008; Wieser et al., 2008), but since we saw no difference in leaf N (Figure 1B) or C:N ratio (not shown) with CO_2_ there is no evidence of growth dilution. While some of the difference between leaf and canopy level responses may be explained by the lower light levels for the measurement of CER relative to that used for the leaf photosynthesis (300 vs. 1000 μmol PAR s^−1^ m^−2^), the relationship of leaf N concentration and canopy CER suggests that there are fundamental differences in photosynthetic N use between plants grown in different soils.

**Figure 7:**
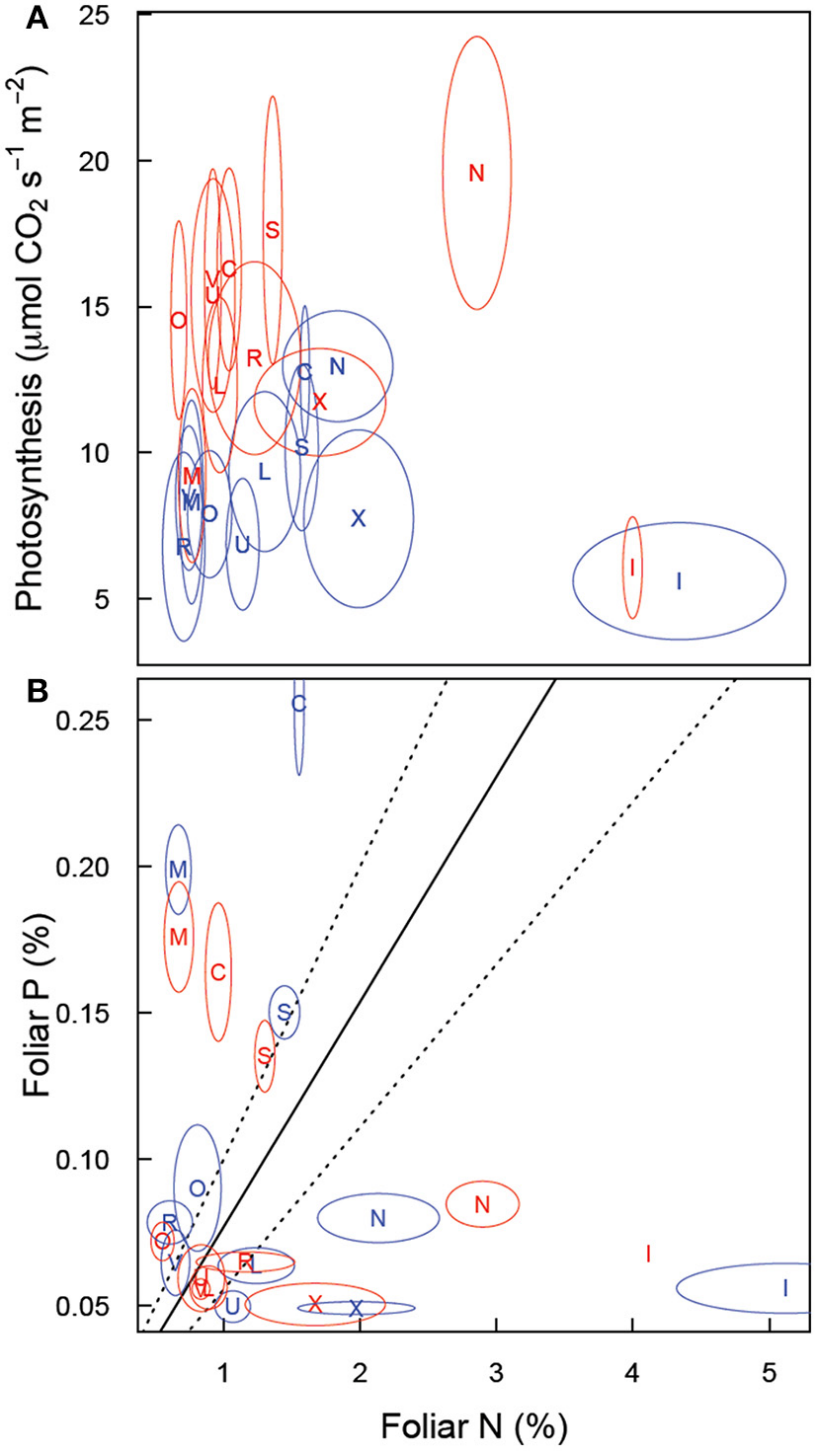
**Relationship of (A) leaf photosynthesis (light saturated, on a leaf area basis) and leaf nitrogen concentration and (B) leaf phosphorus and leaf nitrogen concentration in *Festuca arundinacea* grown in 10 soils, plus a high-fertility control under elevated (800 ppm; red) and ambient (400 ppm; blue) atmospheric CO_2_**. Mean values for each soil × CO_2_ treatment are shown with an ellipse indicating 1 standard error in each dimension. Soil indicated as follows: L, ALF; N, AND; R, ARD; C, CTR; I, INC1; M, MOL; O, OXI1; X, OXI3; S, SPO; U, ULT; V, VRT.

In a review of research on N:P ratios, Güsewell (2004) reported that N:P ratios near 13 were typical for plants grown in their native conditions. In our study, only ALF, ULT and VRT under elevated CO_2_ showed values near this (Figure 7B). A range of 9–19 for N:P ratios was also reported for a range of plants in a range of vegetative communities (Güsewell, 2004), with a range of 10–14 for graminoids. As shown in Figure 7B, few of our plants fell within this range (ALF, ARD, and ULT in elevated CO_2_, and VRT in both atmospheres). Without more detail on the soil the reported N:P ratios represent it is difficult to interpret these results—it is possible that the range of N:P ratios reported does not represent the range of soil variability we are testing here. Alternatively, the wide range of N:P ratios we report here may indicate that fescue is not well adapted to some of these soils. However, correlation between the divergence of observed N:P ratio from the “optimum” value of 13 and biomass was very low (*r* = −0.25) suggests that divergence of N:P ratio from some optimum is not strongly related to biomass.

The progressive nitrogen limitation hypothesis (Luo et al., 2004) suggests that increased plant biomass (and hence soil organic matter) stimulated by elevated CO_2_ can immobilize sufficient N to lead to increasing N limitation with elevated CO_2_. We saw little support for this in this study. Foliar N had limited influence on A_max_ (Figure 7A). Furthermore, biomass was stimulated in only 3 soils (Figure 1A) and root respiration was only modestly increased in four soils (of which two showed above-ground biomass increase also). So in 6 of the 11 soils for which we could fully analyze results there was no stimulation of biomass or below-ground respiration. We note however that the progressive nitrogen limitation hypothesis is likely to operate more strongly on ecosystem spatial scales and over multiple seasons, as a key mechanism for progressive limitation is that a greater proportion of N ends up in plant tissue and in soil organic matter. In this study, the elevated CO_2_ treatment was confined to 10 weeks, which is very unlikely to be a sufficient time-span for progressive limitation to occur to a notable extent.

A substantial number of studies have considered the effect of elevated CO_2_ on root:shoot allocation. We did not harvest roots in this study because the relatively small sizes of plants in this study mean that roots will mostly be rather fine. This fact, combined with the wide range of soil textures (Table 1) would not have yielded reliable data, as the recovery rate for roots would have varied rather strongly with soil texture.

An increase in non-structural carbohydrates (NSC) as been reported in plants exposed to elevated CO_2_ (Poorter and Perez-Soba, 2001; Ziska and Bunce, 2006, 2007). The pattern of accumulation of NSC we observed suggests that carbohydrate physiology may be strongly influenced by differences in soils, though the variability in NSC was rather high (Figure 5). Accumulation of NSC in elevated CO_2_ in the high-fertility control was not significant, while in ALF and AND there was accumulation of both high- and low- weight NSC. In ULT, ARD, and MOL only high weight NSC accumulated. In contrast, for SPO high weight NSC were *reduced* in elevated CO2. The lack of accumulation in SPO, OXI3 and VRT suggests active use of carbon in the plant, possibly in high metabolic demand processes such as mineral acquisition. Alternatively, carbon losses could take place through respiration or rhizo-deposition (Nguyen, 2003). These contrasting responses highlight the ways in which carbohydrate assimilation and metabolism may be influenced by soil conditions.

The four principal components we found in this study improve our understanding of the inter-relationships among mineral content in soils, foliar concentration of minerals, photosynthesis and biomass (Figure 4). In the soils we sampled, foliar levels of Zn, Cu, Mg, Ca, and Na tended to be higher in soils with lower clay content (PC1). In general cations such as Mg and Ca are more available in soils with greater cation exchange capacity, which tend to be soils with higher clay and organic matter content. However, here we see a tendency for lower soil clay to be associated with higher values of these minerals in leaves, suggesting that soil availability of these minerals is not the strongest determinant of their foliar concentration. Spodosols were differentiated on PC1, likely due to the high concentration of calcium and sodium observed in leaves and the low clay content. The high Na content in SPO may have reduced K availability and altered carbohydrate physiology as reflected in the distinct NSC patterns in SPO discussed above. In most soils we observed a decline in the values of PC1 with elevated CO_2_, suggesting a trend of dilution of minerals as was observed clearly with CTR, ALF and ULT. The risk of mineral dilution and the consequent loss of food and forage quality has been mentioned by others (Idso and Idso, 2001; Wieser et al., 2008); our findings suggest that this may affect plants on some soils differently than on others.

PC2 was most influenced by soil minerals (Cu, Ca, Zn), soil pH, foliar P, and foliar Mn (with the opposite sign). This is not surprising, as soil pH governs soil mineral availability, and it is well known that in acidic soils low foliar concentrations of P and high concentration of Mn can inhibit growth (Marschner, 1998). On PC3 photosynthesis and stomatal conductance are opposite in sign to foliar Al, P, Fe, and C. This grouping may indicate limitation of growth and photosynthesis by something other than P; in such conditions foliar P might be less correlated to photosynthetic responses. On PC4, the loading of biomass, low molecular weight NSC (sucrose), and foliar C indicate that growth is favored under conditions that favor sucrose, rather than starch, accumulation in leaves. Starch accumulation in leaves is one symptom of severe P deficiency (Marschner, 1998).

The fact that the first four PCs only captured 65% of the variation in the data indicates that the relationships between photosynthesis, leaf mineral content, and soil physical and chemical properties is complex and highly dimensional—a small number of variables will not adequately describe the range of differences seen. The strongest loadings for CO_2_ were on PC6 (4.7% of the variation) and PC12 (2% of the variation), and CO_2_ was also loaded on PC5 (5.4% of variation). This suggests that variability associated with CO_2_ was relatively low in this data set compared with that associated with plant responses to diverse soils. This suggests that more work is needed with highly diverse soils to better map the potential responses of plants to global change variables.

Soil texture and its influence on plant available water has been suggested as a mechanism that mediates differing responses to elevated CO_2_ (Fay et al., 2012a). The interaction of soil texture and elevated CO_2_ via water availability is an important mechanism that requires further investigation. Our methodology in this study did not test these responses as all plants were well watered. In natural systems where water availability is limiting, responses to elevated CO_2_ could be larger than what we observed. However, as noted by Lynch and St. Clair (2004), toxicity of metals such as Mn can be strongly controlled by soil moisture, so it is also possible that increasing soil water could have negative effects on plant growth. Given the importance of this interaction, a more complete exploration of this interaction is clearly needed. In order to avoid artifacts introduced by sieving or mixing soils, such a test would best be achieved using soil monoliths and a method of providing experimental units with the same total water over the growing season.

The differences among soils in the response to elevated CO_2_ suggest some caution in predicting plant responses to elevated CO_2_ based on the world-wide network of free-air carbon enrichment (FACE) sites without considering how the FACE sites reflect the global diversity of soils. Such caution has been suggested by others who have noted that the distribution of soils limited by acidity (von Uexkull and Mutert, 1995) and phosphorus deficiency (Sanchez, 1976, 1981; Fairhurst et al., 1999; Jaramillo, 2011) and how these contrast with the geographic concentration of the free-air concentration enrichment (FACE) studies in countries in zones free of these edaphic limitations (Schimel, 2006). These differences also raise the possibility that models derived from FACE studies on high fertility sites could be overestimating any positive “silver-lining” effect of climate change on food production (Reilly and Schimmelpfennig, 1999; Long et al., 2006; Leakey et al., 2012).

In this study elevated CO_2_ increased biomass of *Festuca* in only ULT, ALF, and in the high-fertilty control. ULT and ALF are only present in relatively small areas of the world (Table 3), accounting for 17–25% of land area, depending on continent. Others have noted that CO_2_ enrichment studies have predominantly reflected temperate biomes, which may respond differently than do tropical or arctic biomes (Leakey et al., 2012). Since Fescue did not germinate on the Gelisols we cannot speculate on the possible response of plants growing in regions from the tundra and other areas with permafrost soils. These frigid zones are the ones that could experience faster and greater impact of the expected temperature increase due to global change (IPCC, 2007a,b). While we do not claim that the samples utilized in this experiment represent the range of characteristics within each soil order, the soil orders that produced plants with small biomass and with reductions in their carbon assimilation under elevated CO_2_, occupy about 80% of the agricultural area in the world (including grasslands). This highlights the urgent need to better understand the real effects of climate change on plant growth across a representative range of soils, and its implications for food production and ecosystem management.

**Table 3:** **Percent area of each of the soil orders in six continents**.

**Order**	**S. America**	**Africa**	**Asia**	**Oceania**	**N. America**	**Europe**
Alfisols	**10.2**	**12.3**	**4.90**	**14.8**	**10.4**	**25.4**
Andisols	1.39	0.16	0.57	0.88	4.53	0.46
Aridisols	8.22	14.0	11.8	35.1	7.89	0.85
Entisols	15.0	41.8	11.3	26.3	6.89	6.27
Gelisols	0.49	0.00	27.4	0.00	13.3	5.84
Histosols	0.25	0.06	1.58	0.02	2.39	2.68
Inceptisols	11.8	6.36	26.1	4.19	25.5	19.0
Molisols	6.43	0.35	7.75	1.65	11.0	14.5
Oxisols	30.9	13.9	0.21	1.15	0.56	0.00
Spodosols	0.16	0.00	0.71	0.93	11.0	24.2
Ultisols	**14.2**	**7.35**	**6.48**	**3.56**	**4.30**	**0.04**
Vertisols	0.93	3.64	1.30	11.5	2.25	0.77

*Soils in which biomass of Festuca was stimulated by elevated CO_2_ are indicated in bold*.

While we acknowledge the limitations in our study in the use of only one plant species, small pots, and the lack of water stress treatments, the differences between soils in the response to elevated CO_2_ and the importance of soil variables in explaining these differences suggests that a more nuanced consideration of the consequences of soil variability on plant responses to global change may be warranted. The need for studies that address multiple climate change variables and their interactions, particularly with soil, has been noted by others (Lynch and St. Clair, 2004, 2010; Körner, 2006). This study suggests that the variability among soils might be quite large. This points to the need for a substantial research effort to better characterize the interactions of the wide range of soil variability with global change variables. Such an effort should: (1) include not only elevated CO_2_, but water deficit and nitrogen deposition as well; (2) include multiple representatives for each soil order; (3) use intact soil monoliths to assess soils in a more natural context; (4) include more than one species of plant, to address the issue of plant adaptation to specific soils. Furthermore, the methodological challenge of accurately estimating root biomass and length for plants grown in widely differing soils needs to be solved.

### Conflict of interest statement

The authors declare that the research was conducted in the absence of any commercial or financial relationships that could be construed as a potential conflict of interest.

## References

[B1] Bartholome E. Belward A. S. (2005). GLC2000: a new approach to global land cover mapping from earth observation data. Int. J. Remote Sens. 26, 1959–1977 10.1080/01431160412331291297

[B2] Bassirirad H. Gutschick V. P. Lussenhop J. (2001). Root system adjustments: regulation of plant nutrient uptake and growth responses to elevated CO_2_. Oecologia 126, 305–320 10.1007/s004420000524 28547443

[B3] Bucher-Wallin I. K. Sonnleitner M. A. Egli P. Gunthardt-Goerg M. S. Tarjan D. Schulin R. (2000). Effects of elevated CO_2_, increased nitrogen deposition and soil on evapotranspiration and water use efficiency of spruce-beech model ecosystems. Phyton Ann. Rei Bot. 40, 49–60.

[B4] Bunce J. A. (2004). Carbon dioxide effects on stomatal responses to the environment and water use by crops under field conditions. Oecologia 140, 1–10. 10.1007/s00442-003-1401-6 14557864

[B5] Cordain L. (1999). Cereal Grains: humanity's double-edged sword, in Evolutionary Aspects of Nutrition and Health. Diet, Exercise, Genetics and Chronic Disease. World Rev Nutr Diet, ed Simopoulos A. P. (Basel: Karger), 19–73 10.1159/000059677 10489816

[B6] Diaz S. Grime J. P. Harris J. McPherson E. (1993). Evidence of a feedback mechanism limiting plant-response to elevated carbon-dioxide. Nature 364, 616–617 10.1038/364616a0

[B7] Driessen P. M. Konijn N. T. (1992). Land-use systems analysis. Wageningen: Department of Soil Science and Geology, Wageningen Agricultural University.

[B8] Egli P. Maurer S. Spinnler D. Landolt W. Gunthardt-Georg M. S. Körner C. (2001). Downward adjustment of carbon fluxes at the biochemical, leaf, and ecosystem scale in beech-spruce model communities exposed to long-term atmospheric CO_2_ enrichment. Oikos 92, 279–290 10.1034/j.1600-0706.2001.920210.x

[B9] Eguchi N. Morii N. Ueda T. Funada R. Takagi K. Hiura T.. (2008). Changes in petiole hydraulic properties and leaf water flow in birch and oak saplings in a CO_2_-enriched atmosphere. Tree Physiol. 28, 287–295 10.1093/treephys/28.2.287 18055439

[B10] Fairhurst T. Lefroy B. Mutert E. Batjes N. H. (1999). The importance, distribution and causes of phosphorus deficiency as a constraint to crop production in the tropics. Agrofor. Forum 9, 2–8.

[B11] Fay P. A. Jin V. L. Way D. A. Potter K. N. Gill R. A. Jackson R. B. (2012a). Soil-mediated effects of subambient to increased carbon dioxide on grassland productivity. Nat. Clim. Change 2, 742–746 10.1038/nclimate1573

[B12] Fay P. A. Kelley A. M. Procter A. C. Hui D. Jin V. L. Jackson R. B. (2009). Primary productivity and water balance of grassland vegetation on three soils in a continuous CO_2_ gradient: initial results from the lysimeter CO_2_ gradient experiment. Ecosystems 12, 699–714 10.1007/s10021-009-9247-3

[B13] Fay P. A. Polley H. W. Jin V. L. Aspinwall M. J. (2012b). Productivity of well-watered Panicum virgatum does not increase with CO_2_ enrichment. J. Plant Ecol. 5, 366–375 10.1093/jpe/rts007

[B14] Gifford R. M. (2004). The CO_2_ fertilising effect - does it occur in the real world? The international free air CO_2_ enrichment (FACE) workshop: short- and long-term effects of elevated atmospheric CO_2_ on managed ecosystems, Ascona, Switzerland, March 2004. New Phytol. 163, 221–225 10.1111/j.1469-8137.2004.01133.x 33873623

[B15] Gifford R. M. Barrett D. J. Lutze J. L. (2000). The effects of elevated CO_2_ on the C: N and C: P mass ratios of plant tissues. Plant Soil 224, 1–14. 10.1023/A:100479061263014986095

[B16] Güsewell S. (2004). N:P ratios in terrestrial plants: variation and functional significance. New Phytol. 164, 243–266 10.1111/j.1469-8137.2004.01192.x 33873556

[B58] Hagedorn F. Spinnler D. Bundt M. Blaser P. Siegwolf R. (2003). The input and fate of new C in two forest soils under elevated CO_2_. Glob. Change Biol. 9, 862–872 10.1046/j.1365-2486.2003.00638.x

[B17] Heck W. W. Philbeck R. B. Dunning J. A. (1975). A Continuous Stirred Tank Reactor (CSTR) System for Exposing Plants to Gaseous Contaminants: Principles, Specifications, Construction and Operation. New Orleans, LA: USDA-ARS.

[B59] Hendrix D. L. (1993). Rapid extraction and analysis of nonstructural carbohydrates in plant tissues. Crop Sci. 33, 1306–1311.

[B18] Hothorn T. Bretz F. Westfall P. (2008). Simultaneous inference in general parametric models. Biom. J. 50, 346–363. 10.1002/bimj.200810425 18481363

[B19] Idso S. B. Idso K. E. (2001). Effects of atmospheric CO_2_ enrichment on plant constituents related to animal and human health. Environ. Exp. Bot. 45, 179–199. 10.1016/S0098-8472(00)00091-5 11275225

[B20] IPCC. (2007a). Climate Change 2007: Impacts, Adaptation and Vulnerability. Contribution of Working Group II to the Fourth Assessment. Cambridge: Cambridge University Press.

[B21] IPCC. (2007b). Climate Change 2007: The Physical Science Basis. Contribution of Working Group I to the Fourth Assessment. Cambridge; New York, NY: Cambridge University Press.

[B22] Jaramillo R. E. (2011). The Edaphic Control of Plant Response to Climate Change: Extent, Interactions and Mechanisms of Plant Adaptation. Ph.D. thesis, The Pennsylvania State University, University Park, PA.

[B23] Körner C. (2006). Plant CO_2_ responses: an issue of definition, time and resource supply. New Phytol. 172, 393–411. 10.1111/j.1469-8137.2006.01886.x 17083672

[B24] Lambin E. F. Geist H. J. (eds.). (2006). Land-Use and Land-Cover Change. Local processes and Global Impacts. Berlin: Springer-Verlag.

[B25] Leakey A. D. B. Bishop K. A. Ainsworth E. A. (2012). A multi-biome gap in understanding of crop and ecosystem responses to elevated CO_2_. Curr. Opin. Plant Biol. 15, 228–236. 10.1016/j.pbi.2012.01.009 22284851

[B26] Long S. P. Ainsworth E. A. Leakey A. D. B. Nosberger J. Ort D. R. (2006). Food for thought: lower-than-expected crop yield stimulation with rising CO_2_ concentrations. Science 312, 1918–1921. 10.1126/science.1114722 16809532

[B27] Luo Y. Field C. B. Mooney H. A. (1994). Predicting responses of photosynthesis and root fraction to elevated [CO_2_]a: interactions among carbon, nitrogen, and growth. Plant Cell Environ. 17, 1195–1204 10.1111/j.1365-3040.1994.tb02017.x

[B28] Luo Y. Su B. Currie W. S. Dukes J. S. Finzi A. Hartwig U.. (2004). Progressive nitrogen limitation of ecosystem responses to rising atmospheric carbon dioxide. Bioscience 54, 731–739. 10.1641/0006-3568(2004)054[0731:PNLOER]2.0.CO;216634296

[B29] Lynch J. P. St. Clair S. B. (2004). Mineral stress: the missing link in understanding how global climate change will affect plants in real world soils. Field Crops Res. 90, 101–115 10.1016/j.fcr.2004.07.008

[B30] Lynch J. P. St. Clair S. B. (2010). The opening of Pandora's Box: climate change impacts on soil fertility and crop nutrition in developing countries. Plant and Soil 335, 101–115 10.1007/s11104-010-0328-z

[B31] Malinowski D. P. Alloush G. A. Belesky D. P. (2000). Leaf endophyte Neotyphodium coenophialum modifies mineral uptake in tall fescue. Plant and Soil 227, 115–126 10.1023/A:1026518828237

[B32] Malinowski D. P. Belesky D. P. (2000). Adaptations of endophyte-infected cool-season grasses to environmental stresses: mechanisms of drought and mineral stress tolerance. Crop Sci. 40, 923–940 10.2135/cropsci2000.404923x

[B33] Marschner H. (1998). Mineral Nutrition of Higher Plants. London, San Diego: Academic Press.

[B34] Miller O. R. (1998). Microwave digestion of plant tissue in a closed vessel, in Handbook and Reference Methods for Plant Analysis, ed Kalra Y. P. (New York, NY: CRC Press), 69.

[B35] Nguyen C. (2003). Rhizodeposition of organic C by plants: mechanisms and controls. Agronomie 23, 375–396 10.1051/agro:2003011

[B36] Pinheiro J. Bates D. DebRoy S. Sarkar D. R Core Team. (2014). nlme: Linear and Nonlinear Mixed Effects Models. R package version 3.1-117. Available online at: http://CRAN.R-project.org/package=nlme

[B37] Polley H. W. Jin V. L. Fay P. A. (2012). CO_2_-caused change in plant species composition rivals the shift in vegetation between mid-grass and tallgrass prairies. Glob. Change Biol. 18, 700–710 10.1111/j.1365-2486.2011.02529.x

[B38] Poorter H. Perez-Soba M. (2001). The growth response of plants to elevated CO_2_ under non-optimal environmental conditions. Oecologia 129, 1–20 10.1007/s004420100736 28547056

[B39] R Core Team. (2014). R: A Language and Environment for Statistical Computing. Vienna: R Foundation for Statistical Computing Available online at: http://www.R-project.org/

[B40] Rahman M. H. Saiga S. (2007). Endophyte effects on nutrient acquisition in tall fescue grown in andisols. J. Plant Nutr. 30, 2141–2158 10.1080/01904160701700632

[B41] Reilly J. M. Schimmelpfennig D. (1999). Agricultural impact assessment, vulnerability, and the scope for adaptation. Clim. Change 43, 745–788 10.1023/A:1005553518621

[B42] Sanchez P. A. (1976). Properties and Management of Soils in the Tropics. New York, NY: John Wiley.

[B43] Sanchez P. A. (1981). Low-input technology for managing oxisols and ultisols in tropical america. Adv. Agron. 34, 279 10.1016/S0065-2113(08)60889-5

[B44] Schimel D. (2006). Climate change and crop yields: beyond Cassandra. Science 312, 1889–1890. 10.1126/science.1129913 16809520

[B45] Soil Survey Staff. (1999). Soil Taxonomy. Washington, DC: USDA-Natural Resources Conservation Service.

[B46] Sonnleitner M. A. Gunthardt-Goerg M. S. Bucher-Wallin I. K. Attinger W. Reis S. Schulin R. (2001). Influence of soil type on the effects of elevated atmospheric CO_2_ and N deposition on the water balance and growth of a young spruce and beech forest. Water Air Soil Poll. 126 271–290 10.1023/A:1005244916109

[B47] Spinnler D. Egli P. Körner C. (2002). Four-year growth dynamics of beech-spruce model ecosystems under CO_2_; enrichment on two different forest soils. Trees 16, 423–436 10.1007/s00468-002-0179-1

[B57] Spinnler D. Egli P. Körner C. (2003). Provenance effects and allometry in beech and spruce under elevated CO_2_ and nitrogen on two different forest soils. Basic Appl. Ecol. 4, 467–478 10.1078/1439-1791-00175

[B48] Taub D. R. Wang X. (2008). Why are nitrogen concentrations in plant tissues lower under elevated CO_2_? A critical examination of the hypotheses. J. Integr. Plant Biol. 50, 1365–1374. 10.1111/j.1744-7909.2008.00754.x 19017124

[B49] van Noordwijk M. Martikainen P. Bottner P. Cuevas E. Rouland C. Dhillion S. S. (1998). Global change and root function. Glob. Change Biol. 4, 759–772 10.1046/j.1365-2486.1998.00192.x

[B50] von Uexkull H. R. Mutert E. (1995). Global extent, development and economic-impact of acid soils. Plant Soil 171, 1–15 10.1007/BF00009558

[B51] Watanabe M. Mao Q. Z. Novriyanti E. Kita K. Takagi K. Satoh F.. (2013). Elevated CO_2_ enhances the growth of hybrid larch F-1 (*Larix gmelinii* var. japonica x L-kaempferi) seedlings and changes its biomass allocation. Trees 27, 1647–1655. 10.1007/s00468-013-0912-y21813517

[B52] Wieser H. Manderscheid R. Erbs M. Weigel H. J. (2008). Effects of elevated atmospheric CO_2_ concentrations on the quantitative protein composition of wheat grain. J. Agric. Food Chem. 56, 6531–6535. 10.1021/jf8008603 18598044

[B53] Wilding L. P. (2000). Classification of soils, in Handbook of Soil Science, ed Sumner M. E. (Boca Raton, FL: CRC Press), 175–183.

[B54] Woodward F. I. Thompson G. B. McKee I. F. (1991). The effects of elevated concentrations of carbon-dioxide on individual plants, populations, communities and ecosystems. Ann. Bot. 67, 23–38.

[B55] Ziska L. H. Bunce J. A. (2006). Plant responses to rising atmospheric carbon dioxide, in Plant Growth and Climate Change, eds Morison J. I. L. Morecroft M. D. (Oxford, UK: Blackwell Publishing).

[B56] Ziska L. H. Bunce J. A. (2007). Predicting the impact of changing CO_2_ on crop yields: some thoughts on food. New Phytol. 175, 607–617. 10.1111/j.1469-8137.2007.02180.x 17688578

