# Supplementary material for: Response to elevated CO2 in the temperate C3 grass Festuca arundinaceae across a wide range of soils
Source: Front Plant Sci. 2015 Feb 24;6:95. doi: 10.3389/fpls.2015.00095 (PMC4338673; doi:10.3389/fpls.2015.00095)
Supplement: Supplementary file 1 [file Table1.PDF]

Supplemental Information

Response to elevated CO_2 in the temperate C3 grass *Festuca arundinaceae* across a wide range of soils.

Eric A. Nord, Raúl E. Jaramillo, and Jonathan P. Lynch

Table S1. Principal characteristics and areas for the 12 orders of USDA Soil Taxonomy system.

Order	Description	Area (%)*
Alfisols	Soils from semi-arid regions with subsurface accumulation of clay	9.6
Andisols	Developed from volcanic ash	0.7
Aridisols	Desert soils without water for plants	12
Entisols	Soils with minimal evolution, as in eroded or accumulation regions. No subsurface horizons	16.2
Gelisols	Permafrost (frozen soil) within 100 cm from [illegible]	8.6
Histosols	Organic rich, generally in cold latitudes	1.2
Inceptisol	Soils with weakly developed subsurface [illegible]	9.8
Mollisols	Thick dark surface horizon	6.9
Oxisols	soils from tropical regions, highly weathered, deep and uniform profiles	7.5
Spodosols	Bleached horizon over gray-brown (spodic) [illegible]	2.5
Ultisols	Evolved soil with low base saturation (low fertility)in subsurface soil	8.5
Vertisols	Shrink and swell soils, that is soils that exhibit volume temporal variability	2.4

*Percentage of the total land area (1.3 x 10^8 km^2); rows sum to 86% - the remainder (about 14%) is from rock and ice covered regions (Wilding, 2000).

Table S2. Summary of literature search in the Thomson ISI Web of Science (performed in August 2013).

**Results**	**Search Terms**
7,409	FAO soil orders[†]
6,003	US Taxonomy soil orders‡
7,270	"Elevated CO_2"
2,660	"Elevated CO_2" AND soil*
48	"Elevated CO_2" AND soil type
14	"Elevated CO_2" AND edaphic*
6	"Elevated CO_2" and any FAO soil order †
11	"Elevated CO2" and any US Soil taxonomy order ‡

† FAO orders = any of: Acrisol, Andosol, Arenosol, Cambisol, Chernozem, Ferralsol, Fluvisol, Gleysol, Greyzem, Gypsisol, Histosol, Kastanozem, Lithosol, Luvisol, Nitosol, Phaeozem, Planosol, Podzol, Podzoluvisol, Ranker, Regosol, Rendzina, Solonchak, Solonetz, Vertisol, Yermosol

‡ US Soil Taxonomy order = any of: Oxisol, Andisol, Vertisol, Alfisol, Gelisol, Inceptisol, Mollisol, Spodosol, Histosol, Entisol, Ultisol, Aridisol

Table S3. Analysis of topic and methods from 57 unique publication returned by searching "Elevated CO2 AND soil type" and "Elevated CO2 AND (any USDA soil order)" on Web of Science.

**Category**	**Number of Publications**	**Publication Numbers**
Focus on soil responses (C, N, microbial processes), not plant growth	11	3,4,5,12,14,17,41,47,49,50,56
No CO2 treatments	9	1,11,17,22,23,26,48,51,57
Simulation or meta-analysis	8	10,15,18,20,21,32,36,37
Only one soil used	9	8,9,13,23,38,51,52,53,55
Review/summary	1	24
2 soils + elevated CO2	17	2,19,25,27,28,29,30,31,33,34,35,39,40,42,43,44,45
3 soils + elevated CO2	4	6,7,16,54
No experimental data in abstract	1	46

Only 21 of these publications report results from an experiment with two or more soils and elevated CO2, the maximum was three soils, and these 21 reports report on only 6 separate experiments. This highlights the fact that there has been no attempt to consider plant responses to Elevated CO2 across the range of natural soils.

Table S4. Bibliographic data for the publications analyzed in Table S1.

Listing of Publications

1	Bioenergy research, 2014, 7(2)540-550; 10.1007/s12155-014-9417-9
2	Trees-structure and function, 2013, 27(6)1647-1655; 10.1007/s00468-013-0912-y
3	Global change biology, 2013, 19(5)1562-1571; 10.1111/gcb.12140
4	Soil biology & biochemistry, 2013, 58 172-180; DI 10.1016/j.soilbio.2012.11.024
5	Chemosphere, 2013, 90(2)729-736; 10.1016/j.chemosphere.2012.09.057
6	Journal of plant ecology, 2012, 5(4)366-375; 10.1093/jpe/rts007
7	Nature climate change, 2012, 2(10)742-746; 10.1038/nclimate1573
8	Nutrient cycling in agroecosystems, 2012, 93(3)373-385; 10.1007/s10705-012-9523-z
9	Plant production science, 2012, 15(3)238-245
10	Global change biology, 2012, 18(6)2071-2080; 10.1111/j.1365-2486.2012.02650.x
11	Plant and soil, 2011, 349 302-302; 10.1007/s11104-011-0873-0
12	Ecosphere, 2011, 2(8)-; 10.1890/ES11-00117.1
13	Tree physiology, 2011, 31(3)323-338; 10.1093/treephys/tpr001
14	Soil biology & biochemistry, 2011, 43(3)542-550; 10.1016/j.soilbio.2010.11.019
15	Agricultural systems, 2010, 103(3)127-136; 10.1016/j.agsy.2009.11.001
16	Ecosystems, 2009, 12(5)699-714; 10.1007/s10021-009-9247-3
17	Soil biology & biochemistry, 2009, 41(6)1080-1087; 10.1016/j.soilbio.2009.02.013
18	Forest ecology and management, 2008, 255 901-912; 10.1016/j.foreco.2007.09.084
19	Tree physiology, 2008, 28(2)287-295
20	Journal of geophysical research-biogeosciences, 2008, p113; 10.1029/2006JG000270
21	Global change biology, 2007, 13(11)2252-2269; 10.1111/j.1365-2486.2007.01434.x
22	Global change biology, 2007, 13(8)1539-1549; 10.1111/j.1365-2486.2007.01393.x
23	Plant and soil, 2006, 282 83-98; 10.1007/s11104-005-5230-8
24	Grassland: A Global Resource. 20th International Grassland Congress, 2005, Univ Coll Dublin, Ireland, Jun 26-Jul 01, 2005 ISBN 90-769-9871-X 251-264
25	Global change biology, 2003, 9(6)862-872; 10.1046/j.1365-2486.2003.00638.x
26	Eurasian soil science, 2003, 36(2)173-184
27	Basic and applied ecology, 2003, 4(5)467-478; 10.1078/1439-1791-00175
28	Trees-structure and function, 2002, 16(6)423-436; 10.1007/s00468.002.0179.1
29	Oecologia, 2002, 132(1)109-117; 10.1007/s00442-002-0937-1
30	Physiologia plantarum, 2002, 115(2)258-266; 10.1034/j.1399-3054.2002.1150212.x
31	European journal of soil science, 2001, 52(4)619-628; 10.1046/j.1365-2389.2001.00412.x
32	Agriculture ecosystems & environment, 2001, 86(3)221-235; 10.1016/S0167-8809(00)00284-X
33	Water air and soil pollution, 2001, 126 271-290; 10.1023/A:1005244916109
34	Forstwissenschaftliches centralblatt, 2001, 120(1)1-7; 10.1007/BF02796075
35	Oikos, 2001, 92(2)279-290; 10.1034/j.1600-0706.2001.920210.x
36	Ecological modelling, 2000, 134 207-227; 10.1016/S0304-3800(00)00356-2
37	Climate research, 2000, 15(3)221-238; 10.3354/cr015221
38	Global change biology, 2000, 6(5)497-506; 10.1046/j.1365-2486.2000.00333.x
39	Phyton-annales rei botanicae, 2000, 40(4)49-60

40 Functional ecology, 1999, 13(6)748-755; 10.1046/j.1365-2435.1999.00378.x

41 Geoderma, 1999, 90 147-159; 10.1016/S0016-7061(98)00099-8

42 Acta agriculturae scandinavica section b-soil and plant science, 1997, 47(1)14-19; 10.1080/09064719709362433

43 Acta oecologica-international journal of ecology, 1997, 18(3)335-341; 10.1016/S1146-609X(97)80023-9

44 Acta oecologica-international journal of ecology, 1997, 18(3)343-349; 10.1016/S1146-609X(97)80024-0

45 Acta oecologica-international journal of ecology, 1997, 18(3)351-359; 10.1016/S1146-609X(97)80025-2

46 Agricultural and food science in finland, 1996, 5(3)271-283

47 Soil science society of america journal, 1995, 59(5)1321-1328

48 Plant cell and environment, 1993, 16(7)873-878; 10.1111/j.1365-3040.1993.tb00510.x

49 Soil biology & biochemistry, 2013, 65 158-167; 10.1016/j.soilbio.2013.04.021

50 Soil biology & biochemistry, 2013, 57 228-236; 10.1016/j.soilbio.2012.08.024

51 Journal of agronomy and crop science, 2012, 198(6)452-465; 10.1111/j.1439-037X.2012.00516.x

52 Plant and soil, 2012, 358 86-99; 10.1007/s11104-012-1270-z

53 Plant soil and environment, 2012, 58(5)230-235

54 Global change biology, 2012, 18(2)700-710; 10.1111/j.1365-2486.2011.02529.x

55 Agrochimica, 2011, 55(6)314-331

56 Nutrient cycling in agroecosystems, 2011, 89(2)175-187; 10.1007/s10705-010-9386-0

57 Journal of soil science and plant nutrition, 2010, 10(1)12-21; 10.4067/S0718-27912010000100002

Table S5. Loading values of the first four principal components.

	PC1	PC2	PC3	PC4
CO_2	0.0409	-0.0013	0.183	-0.113
Biomass	0.114	0.0869	0.162	**-0.536**
Photosynthesis	-0.0876	0.000688	**0.435**	0.0433
Conductance	-0.0889	-0.0152	**0.35**	-0.00645
Leaf characteristics				
N	-0.213	0.136	-0.0914	0.0583
C	0.0432	-0.0366	-0.286	-0.181
P	-0.0276	**-0.303**	**-0.322**	-0.106
K	0.136	0.213	-0.173	0.0732
Ca	**-0.296**	-0.183	0.00904	-0.0341
Mg	**-0.309**	-0.043	0.0012	0.151
Mn	-0.0897	**0.362**	-0.0441	0.0036
Fe	-0.203	0.2	-0.31	0.00931
Cu	**-0.319**	0.00149	-0.0902	-0.00891
B	-0.188	-0.0309	-0.0649	-0.00341
Al	-0.12	0.14	**-0.385**	0.0554
Zn	**-0.329**	0.0335	-0.0961	-0.0775
Na	-0.269	-0.195	0.101	-0.0547
Soil characteristics				
pH	0.0755	**-0.352**	-0.0196	0.149
P	0.145	-0.24	-0.26	-0.111
K	0.237	-0.0758	-0.166	**-0.33**
Mg	0.133	-0.132	0.106	**0.309**
Ca	0.115	**-0.368**	-0.0153	0.127
Zn	-0.251	-0.258	0.018	**-0.203**
Cu	-0.075	**-0.365**	-0.0181	-0.132
S	-0.00492	0.109	0.108	**-0.53**
Clay %	**0.298**	-0.0802	-0.0696	0.0354
Silt %	0.26	0.111	-0.0545	0.119

Values in boldface are the 5 variables with the greatest absolute loading for each principal component.

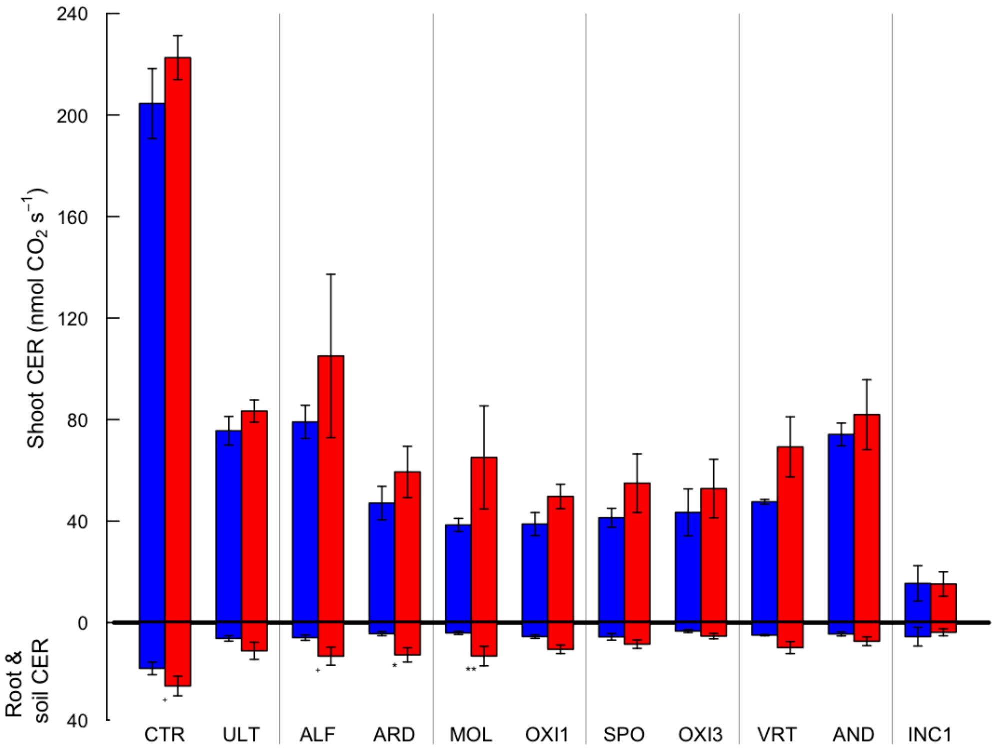

Figure S1. Whole plant shoot (CO_2 uptake) and root (CO_2 evolution) carbon exchange rate (CER) for *Festuca arundinacea* var. Kentucky 31 grown in ambient (400 ppm) and elevated (800 ppm) CO_2 in 10 different soil types and a high-fertility control.

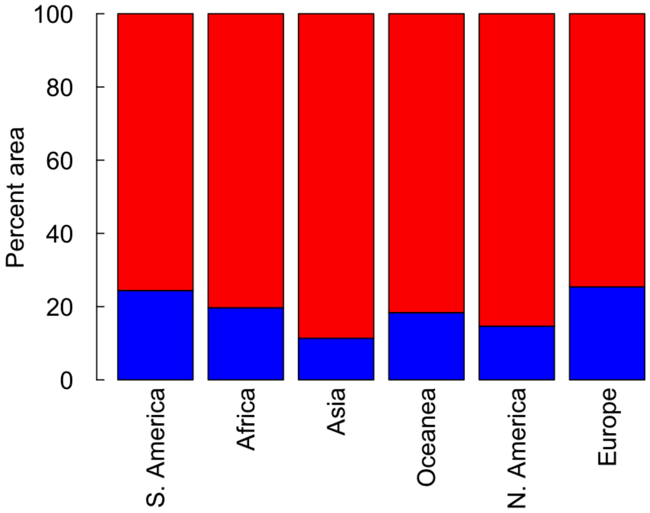

Figure S2. Global distribution of soils classified according to stimulation of *Festuca arundinaceae* growth under elevated CO_2. (Alfisol and Ultisols vs. other soil orders). Dark grey bars represent soils where growth was stimulated, and light grey bars soils where growth was not stimulated.
